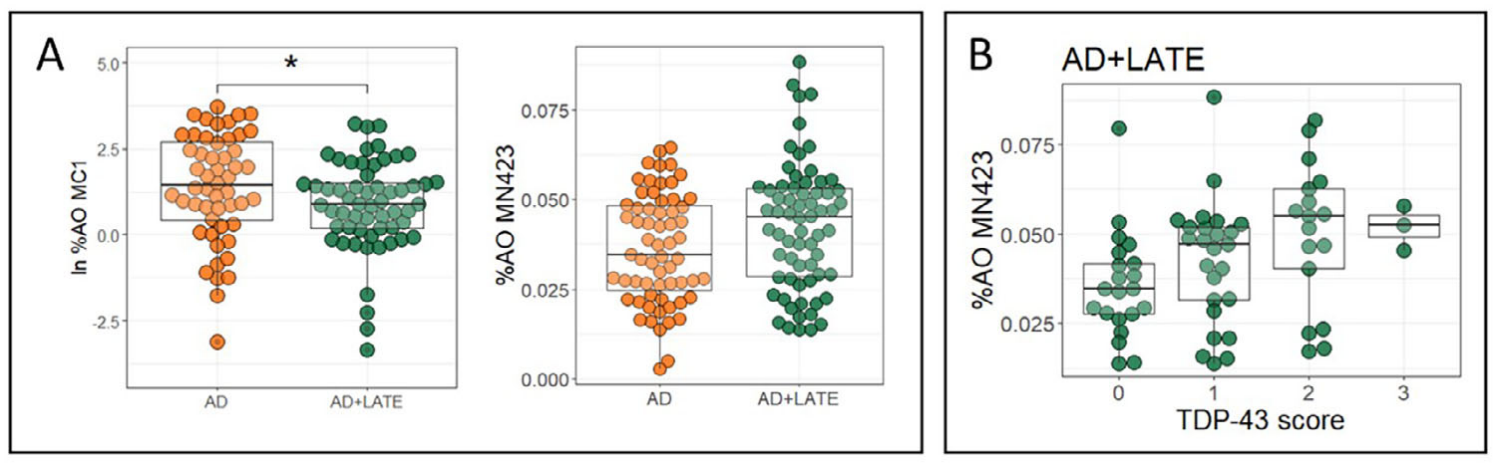# NFT maturation and tau mediated neurodegeneration in Alzheimer’s disease with concomitant LATE

**DOI:** 10.1002/alz.091328

**Published:** 2025-01-03

**Authors:** Sanaz Arezoumandan, Sheina Emrani, Noah Capp, Daniel T Ohm, Jeffrey S Phillips, Corey T McMillan, Eddie B Lee, David A Wolk, David J Irwin

**Affiliations:** ^1^ Department of Neurology, Perelman School of Medicine, University of Pennsylvania, Philadelphia, PA USA; ^2^ Department of Neurology, University of Pennsylvania, Philadelphia, PA USA; ^3^ Department of Pathology & Laboratory Medicine, University of Pennsylvania, Philadelphia, PA USA

## Abstract

**Background:**

Limbic predominant age‐related TDP‐43 encephalopathy (LATE) is a common co‐pathology in Alzheimer’s disease (AD) and is associated with advanced cognitive impairment and severe atrophy in limbic regions. In AD, various maturation stages for tau neurofibrillary tangles have been characterized and can be selectively marked by monoclonal tau antibodies, providing insight into disease progression. Indeed, AD tau pathology progresses from an early “paperclip” conformation, marked by the MC1 epitope to a C‐terminally truncated form of tau, marked by MN423 epitope. Moreover, MC1‐reactive inclusions in AD largely take the form of pre‐tangles/threads and are less prominent in patients with more advanced AD, where there is a higher burden of MN423‐reactive inclusions that largely take the form of extracellular ghost tangles. In this study we tested the hypothesis that hippocampal tau maturation is more advanced in AD with concomitant LATE compared to the pure AD.

**Method:**

Individuals with neuropathological diagnoses of pure AD (N = 63) and AD+LATE (N = 66) were selected. Hippocampal sections were immunohistochemically stained with MC1 and MN423. We measured percent area‐occupied by tau pathology in CA1 of hippocampi by employing validated digital histopathology methods. Additionally, we used ordinal ratings (0‐3) of TDP‐43 pathology from adjacent sections to test the association between tau and TDP‐43 pathology in the AD+LATE group. We compared pathology burden between groups using linear regression models controlling for age, disease duration, sex, and Braak stage.

**Result:**

There were lower levels of early conformations of tau detected by MC1 in the AD+LATE group in contrast to the pure AD group (p = 0.01). Moreover, we found a similar burden of late conformation of tau associated with ghost tangles between the AD+LATE and pure AD group (p = 0.2) (**Fig. 1A**). However, among individuals with AD+LATE, higher TDP‐43 pathology was associated with greater burden of MN423 immunoreactive tau (p = 0.03) (**Fig. 1B**).

**Conclusion:**

The presence of increasing TDP‐43 pathology in AD+LATE is associated with maturation of tau NFT and could potentiate tau‐mediated neurodegeneration.